# Evaluation of Phantom-Based Education System for Acupuncture Manipulation

**DOI:** 10.1371/journal.pone.0117992

**Published:** 2015-02-17

**Authors:** In-Seon Lee, Ye-Seul Lee, Hi-Joon Park, Hyejung Lee, Younbyoung Chae

**Affiliations:** Acupuncture and Meridian Science Research Center, College of Korean Medicine, Kyung Hee University, Seoul, Korea; Tokai University, JAPAN

## Abstract

**Background:**

Although acupuncture manipulation has been regarded as one of the important factors in clinical outcome, it has been difficult to train novice students to become skillful experts due to a lack of adequate educational program and tools.

**Objectives:**

In the present study, we investigated whether newly developed phantom acupoint tools would be useful to practice-naïve acupuncture students for practicing the three different types of acupuncture manipulation to enhance their skills.

**Methods:**

We recruited 12 novice students and had them practice acupuncture manipulations on the phantom acupoint (5% agarose gel). We used the Acusensor 2 and compared their acupuncture manipulation techniques, for which the target criteria were depth and time factors, at acupoint LI11 in the human body before and after 10 training sessions. The outcomes were depth of needle insertion, depth error from target criterion, time of rotating, lifting, and thrusting, time error from target criteria and the time ratio.

**Results:**

After 10 training sessions, the students showed significantly improved outcomes in depth of needle, depth error (rotation, reducing lifting/thrusting), thumb-forward time error, thumb-backward time error (rotation), and lifting time (reinforcing lifting/thrusting).

**Conclusions:**

The phantom acupoint tool could be useful in a phantom-based education program for acupuncture-manipulation training for students. For advanced education programs for acupuncture manipulation, we will need to collect additional information, such as patient responses, acupoint-specific anatomical characteristics, delicate tissue-like modeling, haptic and visual feedback, and data from an acupuncture practice simulator.

## Introduction

Acupuncture manipulation techniques, the heart of acupuncture practice, are regarded as a crucial factor to maximize the effectiveness of acupuncture in clinical practice [1,2]. Recently, many researchers have been trying to conduct standardization and quantification of acupuncture manipulation parameters [3]. Generally, there are two classic kinds of acupuncture manipulation: reinforcing and reducing. Reinforcing, also called tonifying, aims to enrich the *qi* level in the patient’s body, whereas reducing aims to lessen the *qi* level. Most manipulation techniques consist of a combination of the depth of needle insertion, direction of the needle tip, and needle movement to obtain *deqi* (*qi* arrival). After the needle is inserted to the intended depth and direction, rotating (twisting horizontally) and lifting and thrusting (up and down) of the needle are the three basic needle movements. Combining the two basic manipulations and the three basic needle movements provides the manipulations commonly used in acupuncture: rotation (with a clockwise/counterclockwise direction depending on whether it is reinforcing or reducing), reinforcing lifting/thrusting, and reducing lifting/thrusting. Beyond historical research, including studies on an adequate dose of manipulation [4] and standardization of manipulation [5], it is time that more interest be taken in training medical students in manipulation technique.

As acupuncture technique is one of the healing arts, it is difficult to become an expert without training in manipulation skills [6]. Typically, novice students are afraid, at first, of performing acupuncture manipulations on the human body. Skills learned on a variety of surgical simulation models are now known to transfer effectively to the clinical domain [7]. Recently, several studies proposed a virtual-reality training system for acupuncture techniques using a 3D stereo display and realistic haptic feedback in real time [8,9]. Wang *et al*. demonstrated that beginners could imitate, learn, and practice virtual acupuncture by the sensation of needling at acupoints based on the VOXEL-MAN platform [10]. Li *et al*. reported that experts showed more consistent lifting and thrusting technique than novices did when measured using an artificial skin pad [11]. For these virtual acupuncture systems to be implemented appropriately as medical education tools, however, rigorous validation studies are needed to ensure that these devices can mimic haptic sensations during acupuncture manipulations and that they can be useful to enhance the students’ manipulation skills.

We have recently developed and validated an artificial human model based on biomechanical force characteristics similar to human acupoints for acupuncture manipulation training [12]. Our artificial phantom acupoint device has several strong points, such as being inexpensive and portable and providing easy handling of the acupuncture needle. However, there has been little study on whether such phantom acupoint practice can actually improve acupuncture manipulation skills. In this study, we propose a newly developed phantom acupoint as a convenient and helpful tool for training acupuncture-naïve students in manipulations that require much skill. Using the Acusensor 2 device, we evaluated the students’ acupuncture manipulation techniques (target criteria of depth and time factors) at acupoint LI11 in the human body before and after 10 training sessions.

## Materials and Methods

### Participants

The study participants were 12 right-handed students (11 females; age mean = 22.5, standard deviation = 1.7) from the College of Korean Medicine, Kyung Hee University, recruited through poster advertisements on bulletin boards. All participants were 4^th^ grade students and they received their first official practical training in acupuncture in the class of Meridian and Acupoints at Kyung Hee University. The students received a detailed explanation of the study, and written informed consent was obtained. They were informed that no advantage or disadvantage would result from their choosing to participate or not in this experiment and training. All the experiments in this study were conducted in accordance with the guidelines for human subjects committee and approved by the Institutional Review Board of Korea University, Seoul, Republic of Korea.

### Preparation of phantom acupoint

The phantom acupoint was developed and validated in our previous study [12]. In this study, a phantom acupoint made of 5% agarose gel was used with human acupoint LI11 because it produced similar biomechanical force and needle grasp sensation during needle rotation in the previous study [12].

### Pre-defined acupuncture manipulation skills

**Fig 1:**
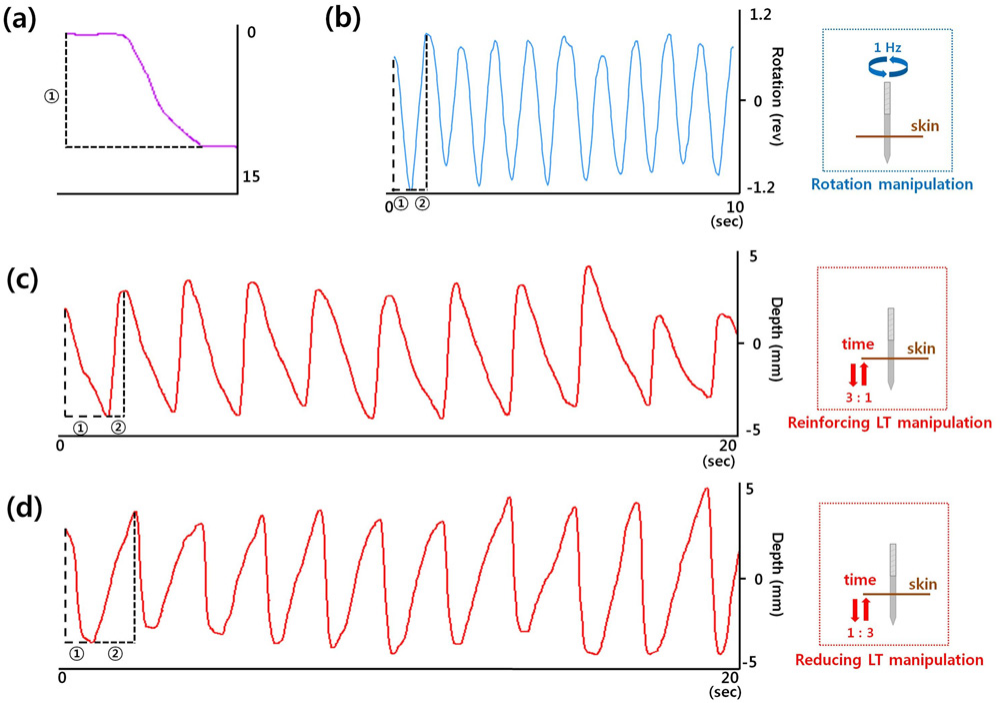
Sample graph showing motion and time factors of three manipulations. (a) Depth of needle (depth of needle insertion, mm), (b) rotation, 10 times for 10 s (thumb-forward time, target criterion 0.5 s; thumb-backward time, target criterion 0.5 s), (c) reinforcing LT, 10 times for 20 s (thrusting time, target criterion 1.5 s; lifting time, target criterion 0.5 s), (d) reducing LT, 10 times for 20 s (thrusting time, target criterion 0.5 s; lifting time, target criterion 1.5 s).

A doctor of Korean medicine (graduate of the College of Korean Medicine, Kyung Hee University, licensed to practice acupuncture) gave a brief lecture introducing the three kinds of acupuncture manipulation (rotation, reinforcing lifting/thrusting (LT), and reducing lifting/thrusting (LT)) and the methods of acupuncture practice on humans and the phantom acupoint (Fig. 1). The exact target depth (15 mm), thumb-forward (TF) time (0.5 s), thumb-backward (TB) time (0.5 s), lifting time (reinforcing LT, 0.5 s; reducing LT, 1.5 s), thrusting time (reinforcing LT, 1.5 s; reducing LT, 0.5 s), ratio of time between motions (rotation, TF time to TB time = 1:1; reinforcing LT, thrusting time to lifting time = 3:1; reducing LT, lifting time to thrusting time = 3:1), and total manipulation time (rotation, 10 s; LT, 20 s) and number (10 times) were described to the students (Table 1).

**Table 1:** Pre-defined motion and time factors of the three manipulations.

(1) Types	(2) Needle depth	(3) TF (rotation) or thrusting time (LT)	(4) TB (rotation) or lifting time (LT)	(5) Ratio of times*	(6) Total manipulation time/number
Rotation	15 mm	0.5 s	0.5 s	1:1	10 s/10
Reinforcing LT	15 mm	0.5 s	1.5 s	3:1	20 s/10
Reducing LT	15 mm	1.5 s	0.5 s	3:1	20 s/10

Rotation: rotating needle thumb forward for 0.5 s and thumb backward for 0.5 s 10 times during 10 s; reinforcing LT: thrusting needle for 0.5 s and lifting for 1.5 s 10 times during 20 s; reducing LT: thrusting needle for 1.5 s and lifting for 0.5 s 10 times during 20 s. The depth of needle was targeted at 15 mm for every manipulation.

*Ratios of times were rotation, TF time to TB time = 1:1; reinforcing LT, thrusting time to lifting time = 3:1; and reducing LT, lifting time to thrusting time = 3:1.

The Acusensor 2 (Stromatec, Inc., VT, USA, www.stromatec.com) was used to record real-time wave data during the three types of manipulation: rotation, reinforcing LT, and reducing LT. With the device’s motion sensor, depth of needle insertion, and time required for each motion (TF and TB during rotation, needle lifting and thrusting during reinforcing LT and reducing LT) were recorded.

### Measurement of acupuncture manipulation skills

On day 1 (D1), the baseline manipulation techniques of the students in the three types of manipulations were measured using the Acusensor 2. Participants were paired and practiced acupuncture manipulation on their partner’s human acupoint (left LI11). After sterilizing the acupoint with alcohol, the motion sensor of the Acusensor 2 was located on the left LI11 of the partner. Acupoint LI11 is located on the lateral aspect of the elbow at the midpoint of the line connecting LU5 with the lateral epicondyle of the humerus. Then, the participant inserted the acupuncture needle (J-type Japanese Seirin needle: 0.25 × 40 mm, Seirin, Japan) through the motion sensor until it was placed lightly on the surface of the skin, though not penetrating the skin, to enable measurement of the depth of insertion. After the Acusensor 2 data-collection software started to record real-time wave data of the needle motion, participants inserted the needle into the skin until it reached the target depth. When participants thought the needle had reached the targeted depth (15 mm), they started the manipulation in a pre-defined sequence (rotation, reinforcing LT, reducing LT). Needle depth and time of each motion (TF, TB, lifting, thrusting) were recorded with the Acusensor 2, but participants were not allowed to check the real-time wave data on the monitor. After the rotation, reinforcing LT, and reducing LT at the human acupoint were finished, the practitioner and partner changed roles.

On day 10 (D10), participants repeated the acupuncture manipulation at acupoint LI11, and data were again recorded using the Acusensor 2 in the same order.

### Procedures for training in acupuncture manipulation skills

After recording their baseline acupuncture manipulation technique, participants received 10 phantom acupoint tubes (5% agarose gel in Safe-lock tubes (2 mL, Eppendorf, Hamburg, Germany)) and a practice diary. They were told to practice using only the 10 phantom acupoint tubes, and the goal of practice was to successfully improve their manipulation technique to meet the target criteria we offered. They were told to practice 10 times over 10 days and to record the number of practice sessions, type of manipulation they practiced, practice methods, and self-assessment of technique improvement using a 0–10 visual analog scale (Question: ‘How much do you think this practice improved your manipulation technique?’; 0: not likely at all, 10: very likely).

### Data processing and analysis

The depth of needle insertion, depth error, time ratio, and time error were calculated as the mean ± standard error (SE) of the 12 participants. The depth error represents the absolute deviation of needle insertion from the target depth (|15 mm—depth of needle|). The time error represents the absolute deviation of time required to needle motion from the target time (|0.5 s—TF, TB time| in rotation, |0.5 s—lifting time|, |1.5 s—thrusting time| in reinforcing LT, |1.5 s—lifting time|, |0.5 s—thrusting time| in reducing LT). The values are presented as the mean ± SE. Data were compared between the D1 (before training) and D10 (after training) using a paired *t*-test. Statistical analyses were performed using the SPSS software (ver. 20.0 for Windows; SPSS, Inc.; Chicago, IL, USA). A *p*-value < 0.05 was considered to indicate statistical significance.

## Results

### Self-assessment of technical skills

During the 10 days, participants practiced the three types of manipulation 9.25 ± 0.56 times for 22.11 ± 2.65 min per practice session. The self-assessment of technical skill was significantly improved after training (D1: 3.00 ± 0.46 vs. D10: 5.44 ± 0.52, *P* < 0.05).

### Measurement of depth in acupuncture manipulation techniques

**Fig 2:**
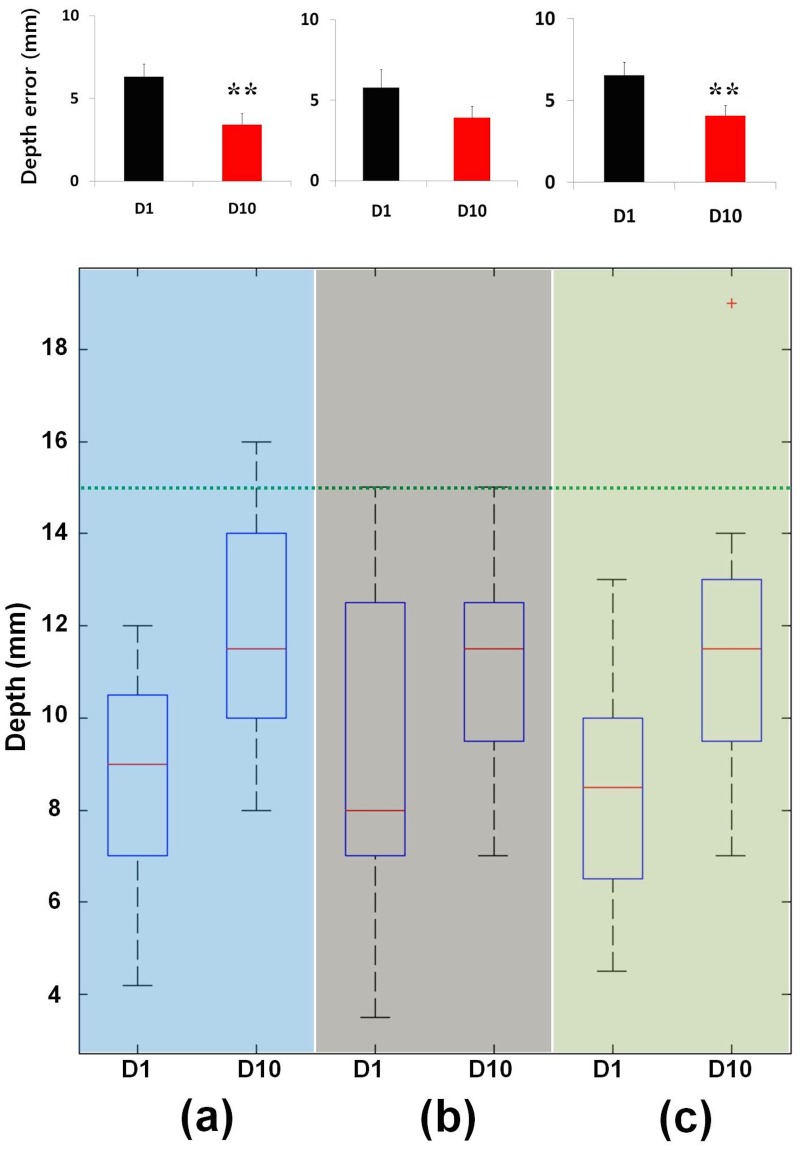
Measurements of depth in acupuncture manipulation techniques. (a) Rotation: the depth error was decreased significantly (*P* < 0.01), and the distribution of depth at D10 was closer to the target depth (green line) than it was at D1. (b) Reinforcing LT; the depth error was decreased, and the distribution of depth at D10 was narrower and closer to the target depth (green line) than it was at D1. (c) Reducing LT; the depth error was significantly decreased (*P* < 0.01), and the distribution of depth at D10 was closer to the target depth (green line) than it was at D1. Depth error = |15 mm—depth of needle|, mean ± SE. The green line indicates the target depth (15 mm). **: *P* < 0.01.

In the rotation manipulation techniques, the depth of needle insertion was significantly increased at D10 versus D1 (target 15 mm, D1: 8.68 ± 0.77, D10: 11.92 ± 0.81 mm, *P* < 0.01). The depth error was significantly decreased at D10 versus D1 (D1: 6.32 ± 0.77, D10: 3.42 ± 0.66 mm, *P* < 0.01). The distribution of depth in D10 was closer to the target depth (15 mm) than was that at D1 (Fig. 2a).

In the reinforcing LT manipulation technique, the depth of needle insertion was increased at D10 versus D1 but not significantly different (target 15 mm, D1: 9.21 ± 1.11, D10: 11.08 ± 2.39 mm, *P* = 0.14). Depth error was decreased at D10 versus D1, but not significantly different (D1: 5.79 ± 1.11, D10: 3.92 ± 0.72 mm, *P* = 0.14). The distribution of depths at D10 was narrower and closer to the target depth (15 mm) compared with that at D1 (Fig. 2b).

In the reducing LT manipulation technique, the depth of needle insertion was significantly increased at D10 versus D1 (target 15 mm, D1: 8.46 ± 0.81, D10: 11.58 ± 0.95 mm, *P* < 0.05). Depth error was significantly decreased at D10 versus D1 (D1: 6.54 ± 0.81, D10: 4.08 ± 0.64 mm, *P* < 0.01). The distribution of depth at D10 was closer to the target depth (15 mm) than was that at D1 (Fig. 2c).

### Time measurements in acupuncture manipulation techniques

**Fig 3:**
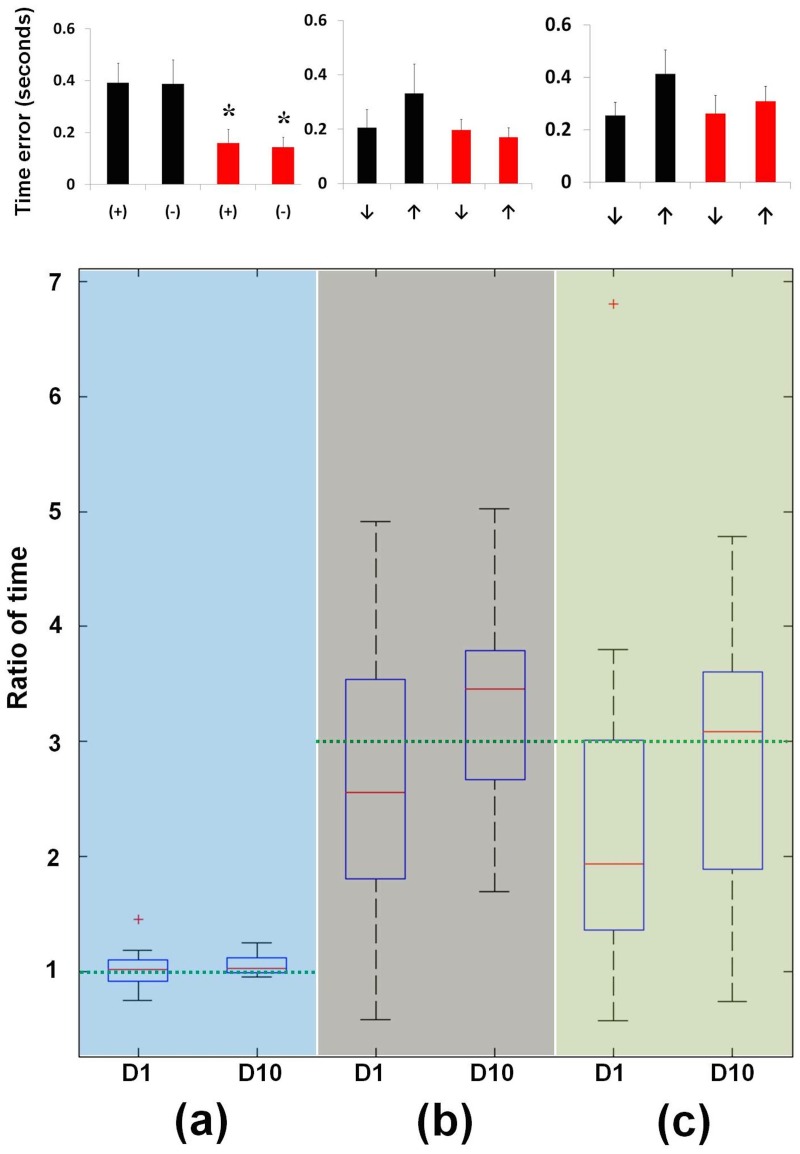
Measurements of time in acupuncture manipulation techniques. (a) Rotation; (+): Thumb forward (TF); (-): Thumb backward (TB); TF time error = |0.5 s—TF time|, mean ± SE; TB time error = |0.5 s—TB time|, mean ± SE; Target ratio of time = TF time/TB time = 1:1. The TF and TB time error was significantly decreased (*P* < 0.05), and the distribution of ratio of times at D10 was narrower than that at D1. (b) Reinforcing LT, ↑: lifting, ↓: thrusting, Lifting time error = |0.5 s—lifting time|, mean ± SE; Thrusting time error = |1.5 s—thrusting time|, mean ± SE; Target ratio of time = thrusting time/lifting time = 3:1. The lifting and thrusting time errors were decreased, and the distribution of the ratio of time at D10 had a narrower distribution than that at D1. (c): Reducing LT, ↑: lifting, ↓: thrusting, Lifting time error = |1.5 s—lifting time|, mean ± SE; Thrusting time error = |0.5 s—thrusting time|, mean ± SE; Target ratio of time = lifting time/thrusting time = 3:1. The lifting time error was decreased, and the thrusting time error was slightly increased. The distribution of the ratio of time at D10 was closer to the target ratio (green line) than that at D1. The green line indicates the target ratio of time (1:1, 3:1, 3:1, respectively).

In the rotation manipulation technique, the TF time was decreased at D10 versus D1, but not significantly different (target 0.5 s, D1: 0.82 ± 0.1, D10: 0.63 ± 0.06 s, *P* = 0.11). The TF time error was significantly decreased at D10 versus D1 (D1: 0.39 ± 0.08, D10: 0.16 ± 0.05 s, *P* < 0.05). The TB time was markedly decreased at D10 versus D1 (target 0.5 s, D1: 0.83 ± 0.11, D10: 0.59 ± 0.05 s, *P* = 0.07). The TB time error was significantly decreased at D10 versus D1 (D1: 0.39 ± 0.09, D10: 0.14 ± 0.04 s, *P* < 0.05). There was no significant difference between the ratio of time (TF time to TB time) at D1 and that at D10 (target ratio 1:1, D1: 1.03 ± 0.06, D10: 1.06 ± 0.03, *P* = 0.64). The distribution of the ratio of time at D10 was narrower than was that at D1 (Fig. 3a).

In the reinforcing LT manipulation technique, lifting time was significantly decreased at D10 versus D1 (target 0.5 s, D1: 0.74 ± 0.13, D10: 0.44 ± 0.06 s, *P* < 0.05). The lifting time error was decreased at D10 versus D1, but not significantly different (D1: 0.33 ± 0.11, D10: 0.17 ± 0.03 s, *P* = 0.14). The thrusting time was decreased at D10 versus D1, but not significantly different (target 1.5 s, D1: 1.45 ± 0.09, D10: 1.32 ± 0.05 s, *P* = 0.29). The thrusting time error was slightly decreased at D10 versus D1 (D1: 0.21 ± 0.07, D10: 0.20 ± 0.04 s, *P* = 0.92). There was a significant difference between the ratio of times (thrusting time to lifting time) at D1 compared with D10 (target ratio 3:1, D1: 2.61 ± 0.39, D10: 3.36 ± 0.31, *P* < 0.05). The distribution of the time ratio at D10 was narrower than that at D1 (Fig. 3b).

In the reducing LT manipulation technique, lifting time was decreased at D10 versus D1, but not significantly different (target 1.5 s, D1: 1.28 ± 0.14, D10: 1.20 ± 0.06 s, *P* = 0.58). The lifting time error was decreased at D10 versus D1, but not significantly different (D1: 0.41 ± 0.09, D10: 0.31 ± 0.06 s, *P* = 0.38). The thrusting time was decreased at D10 versus D1, but not significantly different (target 0.5 s, D1: 0.67 ± 0.08, D10: 0.55 ± 0.11 s, *P* = 0.23).

The thrusting time error was slightly increased at D10 versus D1 (D1: 0.25 ± 0.05, D10: 0.26 ± 0.07 s, *P* = 0.91). There was no significant difference between the ratio of time (lifting time to thrusting time) at D1 compared with D10 (target ratio 3:1, D1: 2.38 ± 0.50, D10: 2.89 ± 0.39, *P* = 0.28). The distribution of the ratio of time at D10 was closer to the target ratio of 3:1 than that at D1 (Fig. 3c).

## Discussion

In the present study, we demonstrated significant improvements in students’ manipulation techniques on the human body through training with the phantom acupoint. After this training, students inserted the needle on a human acupoint closer to 15 mm (the target depth) and successfully reduced depth error. When performing manipulations, the time error decreased after the training, and the ratios of times were closer to the target criteria.

The degree of improvement in manipulation technique varied with the type of manipulation and outcome. Four outcomes improved markedly in rotation manipulation, and two outcomes in reducing LT and one in reinforcing LT improved significantly after the training. According to the post-experiment interview, students usually considered LT techniques more difficult than rotation techniques in our study. Thus, variation in the improvements in different manipulation types might have resulted from the differences in the difficulty of the manipulations. Our findings suggest that education tools and programs could be developed separately for each manipulation technique.

Part of the experiment that should be noted was that the students were not allowed to practice on the human body during the training period. They performed the acupuncture manipulation on a human body when they were tested before and after the training, and during the training session, they used only the phantom acupoint. Thus, it is important to understand how the qualities of a phantom acupoint that are similar to or different from a human acupoint affect the improvement in acupuncture manipulation skills. The interest in so-called phantom-based education has increased in many fields and has already advanced in many ways, such as the application of simulators with haptic and visual feedback [13,14,15]. The main purpose of phantom-based education is to enhance the practical skills with original tools on an original model (acupuncture on the human body, dental treatment on human teeth), not with a phantom tool on a phantom model (acupuncture on a phantom acupoint, dental treatment on artificial teeth). Thus, the most important point to consider when developing a phantom tool is the implementation of the necessary properties of the original. Some properties are crucial in a phantom, whereas some do not necessarily relate to the different functions and characteristics of the original tool.

For a phantom acupoint, for example, safety, size, cost and similarity between the needle-grasp sensation when practicing the manipulation and when working with the human acupoint are critical. Agarose gel, the material used in the phantom acupoint in the present study, is well known for its safety, and its resistance can easily be controlled by modifying the concentration of the gel. However, the most important characteristic is the subjective needle-grasp sensation delivered to the practitioner during manipulation of the acupuncture needle into the acupoint. In our previous study, we developed the phantom acupoint and demonstrated the validity of the phantom acupoint by showing that rotating a needle into the phantom acupoint exhibited biomechanical forces and conveyed needle grasp sensations to the practitioner that were similar to those of a human acupoint [12].

There are several advantages of using the phantom acupoint instead of a human acupoint. As we have mentioned, naïve or unskilled practitioners commonly fear performing acupuncture at first. Experiencing acupuncture practice with the phantom acupoint can help them to feel comfortable throughout the process, from opening an acupuncture package to inserting the needle. Second, as the phantom acupoint is transparent, practitioners can check the depth of needle insertion, unlike a human acupoint, although the depth of the inserted needle can be assessed by the length of the needle that remains outside the skin. Third, the degree of the manipulation (amplitude, frequency, time) could be unlimited unless the phantom acupoint crumbles. Performing acupuncture manipulation on humans usually provides insufficient practice because subjects complain of pain after a few sessions. Finally, if combined with visual feedback (by allowing students to check the real-time data of the Acusensor 2 on the monitor), practice on the phantom acupoint allows students to receive haptic and visual feedback simultaneously. This would help students to perform manipulations more consistently, or differentially, depending on their purpose.

Some suggestions for improvement are as follows. The phantom acupoint could be upgraded to reflect patients’ subjective sensations, such as pain and *deqi*. Furthermore, more precise modeling of human tissue that duplicates the characteristics of each acupoint would provide more realistic appearance and tension. According to pre-experiment interviews with the students, the difficulties in practicing acupuncture, other than manipulation, were finding the appropriate acupoint on the human body, penetrating the skin without pain, varying the depth of the needle depending on different acupoints, and inserting the needle tip in the proper direction. Upgrading the current phantom acupoint considering these issues would be possible by integrating mechanical development and anatomical research on the biomechanical features of acupoints. For example, practicing needle penetration into the skin requires a dense skin layer, and availability of a variety of phantom acupoints would enhance training. In order to broaden the phantom-based education system for acupuncture manipulation in real world, however, it is required to validate the usefulness of this education program in other institutions or other countries based on Good Laboratory Practice criteria [16].

In conclusion, we demonstrated that the phantom acupoint is a convenient and useful training tool to improve the techniques of acupuncture manipulation by measuring depth of needle insertion, absolute times, and ratios of times during needle rotation, reinforcing lifting/thrusting, and reducing lifting/thrusting. Considering patient reactions, acupoint-specific anatomical characteristics, delicate tissue-like modeling, combined with haptic and visual feedback, an acupuncture practice simulator could be developed as an advanced education system for acupuncture skills. It would be interesting to develop the acupuncture manipulation education system based on visuomotor learning. Visual feedback with motion information of students’ hand movements during acupuncture manipulation enables novices to improve their skills through the acquisition of the sensorimotor representation of the observed motion template.
